# Fully Automatic Knee Bone Detection and Segmentation on Three-Dimensional MRI

**DOI:** 10.3390/diagnostics12010123

**Published:** 2022-01-06

**Authors:** Rania Almajalid, Ming Zhang, Juan Shan

**Affiliations:** 1 Department of Computer Science, Seidenberg School of CSIS, Pace University, New York, NY 10038, USA; ra56319p@pace.edu; 2 College of Computing and Informatics, Saudi Electronic University, Riyadh 11673, Saudi Arabia; 3 Department of Computer Science & Networking, Wentworth Institute of Technology, Boston, MA 02115, USA; 4 Division of Rheumatology, Tufts Medical Center, Boston, MA 02111, USA

**Keywords:** knee osteoarthritis, fully automatic bone detection and bone segmentation, 3D MRI, convolutional neural networks, U-net

## Abstract

In the medical sector, three-dimensional (3D) images are commonly used like computed tomography (CT) and magnetic resonance imaging (MRI). The 3D MRI is a non-invasive method of studying the soft-tissue structures in a knee joint for osteoarthritis studies. It can greatly improve the accuracy of segmenting structures such as cartilage, bone marrow lesion, and meniscus by identifying the bone structure first. U-net is a convolutional neural network that was originally designed to segment the biological images with limited training data. The input of the original U-net is a single 2D image and the output is a binary 2D image. In this study, we modified the U-net model to identify the knee bone structures using 3D MRI, which is a sequence of 2D slices. A fully automatic model has been proposed to detect and segment knee bones. The proposed model was trained, tested, and validated using 99 knee MRI cases where each case consists of 160 2D slices for a single knee scan. To evaluate the model’s performance, the similarity, dice coefficient (DICE), and area error metrics were calculated. Separate models were trained using different knee bone components including tibia, femur, patella, as well as a combined model for segmenting all the knee bones. Using the whole MRI sequence (160 slices), the method was able to detect the beginning and ending bone slices first, and then segment the bone structures for all the slices in between. On the testing set, the detection model accomplished 98.79% accuracy and the segmentation model achieved DICE 96.94% and similarity 93.98%. The proposed method outperforms several state-of-the-art methods, i.e., it outperforms U-net by 3.68%, SegNet by 14.45%, and FCN-8 by 2.34%, in terms of DICE score using the same dataset.

## 1. Introduction

Knee osteoarthritis (OA) is a progressive chronic condition described by any changes in the structure of the bone, cartilage, synovium, or other joint structures [1,2,3]. Among the different types of arthritis, OA affects the elderly and is considered to be the most recurrent type. This arthritis condition reduces the lifespan of elderly people as it poses restrictions in carrying out their tasks, which results in not only a disability but also financial constraints for the societies and their healthcare systems. Research conducted in 2000, showed that the group of people aged 65 years and above makes up 13% of the total U.S. population and half of this population suffered from OA in one of their joints [4]. Data analyzed in 2004, revealed that the U.S. spent approximately USD 336 billion, which is equivalent to 3% of its gross domestic product (GDP), to take care of the people infected with arthritis [5]. Furthermore, the total lifetime care cost for anyone infected with knee OA was calculated to be USD 140,300 [6]. In 2010, it was estimated that 9.9 million U.S. adults suffer from symptomatic knee OA [7]. By 2030, an estimation of 20% of the U.S. population, around 70 million persons, will be 65 years old and may be inclined to knee OA [4], posing a great financial burden to society due to the excessive costs incurred in joint replacement. There are also higher chances of one leaving the workforce early as well as increasing the rate of absenteeism from work because of knee OA [8].

Over many years of research, there is improved knowledge about the knee OA like the cause of pain and loss of joints mobility which is the result of degradation of the articular cartilage [9], but the development mechanism and pathogenesis of the knee OA are still unclear including the intervention or remedy that may slow the progress of the ailment [10]. To effectively measure the knee joint, various medical imaging methods such as X-ray, Computed Tomography (CT), and Magnetic Resonance Imaging (MRI) could be used [11]. The MRI can generate high-resolution images to study and understand the soft-tissue structures that include muscles, hyaline cartilage, bone marrow lesion, and meniscus from different perspectives. It is considered the most effective modality for noninvasive examination of the articular cartilage, and deterioration of the cartilage can be easily assessed through MRI [12,13]. One of the challenges encountered in using MRI is that it’s time-consuming to review and analyze MRI since every MRI scan generates 160 slices of 2D images and manual segmentation of cartilage for one 3D MRI knee could take up to six hours. In order to precisely measure and analyze cartilage, comprehensive and vast training is needed [14]. Since the high labor cost makes the diagnosis costly, less time-efficient, and difficult to replicate, the technologies of computer-aided segmentation for the knee MRI are desperately needed [15]. To automatically determine knee structures such as bone marrow lesion, meniscus, cartilage, and muscle from a knee MRI, the segmentation of the bone is an essential first step since the bone occupies the major part in knee MRI and integrates with all other structures.

Scientists have done several investigations using the MR images to get the most appropriate and faster methods in order to measure the knee structures, which include the segmentation of alternate MR slices as well as the limitation of the assessment to partial areas of those structures [16,17,18]. To automatically segment MR images, computer-aided algorithms have been involved based on the active-contour models [16,17,19,20,21]. However, these methods were not robust enough to be used in clinical research, especially in detecting small changes in the structure [18]. Direct segmentation of cartilage without bone recognition is more difficult due to the complexity of its structure. Thus, to make this work easier, the inspiration of identifying the bone first can be served as the first step of segmenting the other structures such as bone marrow and cartilage.

Deep learning (DL) methods have successfully addressed critical problems in the vision and audio fields since they are known for their ability to extract high-level features [22,23,24]. DL methods can produce excellent segmentation and classification results because they have the power to learn directly from the raw high dimensional input and extract its features layer by layer. Nowadays, the interest in DL applications using medical images has been risen [25]. CNNs has demonstrated superior performance in solving many medical image segmentation problems and achieved satisfactory results for different segmentation tasks include mandible segmentation [26], sinonasal cavity and pharyngeal airway segmentation [27], brain segmentation [28], optic disc segmentation [29], liver segmentation [30], lung segmentation [31], etc. Among all different deep learning models, U-net [32] was developed for the segmentation of neuron structure in microscopy images. Its convolutional network has a distinguished “U” shaped architecture. The U-net won the ISBI challenge since it generated fast and precise segmentation results. U-net was adopted by many studies on medical image analysis because of its tolerance with small datasets and the ability to generate robust and accurate segmentation results.

Doctors and radiologists use semi-automatic segmentation methods to perform knee MRI segmentation through human–computer interaction. Semi-automatic segmentation might be achieved by the use of a number of algorithms, like live wire [33], active contours [34,35,36,37,38], ray casting [39,40], region growing [39,40,41], and watershed [42]. In [43], both 3D and 2D deep-learning based segmentations were used with statistical shape models as shape refinement post-processing for tibia and femur bone segmentation independently. In our earlier work [44], a segmentation model for knee femur bones was established using a modified U-net structure. All the slices that had a femur bone were chosen to train and test the model which means all the slices with no bone appearance were excluded. Therefore, the study focused on the segmentation of the femur bone only. In [45], Liu et al., developed a fully automatic segmentation pipeline that combines a deep CNN and 3D simplex deformable modeling. The method performed segmentation of knee tissues which include tibia bone, femur bone, tibia cartilage, and femur cartilage. The 10-layers SegNet was employed as the core of the segmentation pipeline to conduct pixel-by-pixel multi-class classification, using 2D knee images with high resolution. In the testing phase, the generated pixel-wise labels from the SegNet were sent into an iterative processing filter that used a connected-component filter to fill gaps and eliminate tiny isolated items. Then these labels were passed to the marching cube algorithm to build a 3D simplex mesh for each individual segmentation object. The 3D simplex mesh was sent to the 3D simplex deformable model for refining based on the source image. Finally, the method generated 3D segmentation by combining all the deformed objects. The public SKI10 dataset was used to test the proposed segmentation pipeline. In [46], Liu developed a knee joint segmentation method to segment tibia bone, femur bone, tibia cartilage, and femur cartilage using adversarial networks. Since the medical datasets are available in a variety of tissue contrasts, the manual annotation for each sequence can be time-consuming. Thus, the author utilized the cycle-consistent generative adversarial network (CycleGAN) for image translation across MRI datasets with varying tissue contrasts in order to reduce the amount of human effort required for manual segmentation. For segmentation purposes, a Segment Unannotated image Structure using Adversarial Network (SUSAN) was proposed. Regarding the CNN mapping functions, the author developed a method called R-Net which enables dual outputs by bifurcating after the last up-sampling layer in the decoder. To test the proposed system, the public SKI10 dataset has been used with additional two clinical knee MR image datasets acquired from the department of radiology at University of Wisconsin-Madison. One of the clinical datasets consisted of T2-FSE sequence and the other one consisted of PD-FSE sequence. Table 1 presents a summary of the recent knee segmentation methods.

In this work, we extended our earlier study and developed a fully automatic method that can detect MRI slices with knee bone first and then segment bone structures accurately. In addition, the proposed method can handle all femur, tibia, and patella bones no matter they are presented individually or combined. The main difference between this study and previous work [43,44] is that our method does not require any human intervention; the trained model takes the whole MRI sequence (160 slices) as input and outputs all the segmented bone structures in every slice with bone, and slices without bone are discarded automatically through the detection model. The second difference is that previous studies focused on parts of the knee bone structures, either the femur bone only or femur and tibia bones; in this work, our method can detect and segment entire knee bones, which include all tibia, femur, and patella bones.

The rest of the paper is organized as follows. In Section 2, we described our dataset and discussed the proposed method which included the modified structure of U-net, the separation of the training and testing sets as well as the implementation. Section 3, presented and analyzed the experiment results along with the evaluation metrics that have been used while Section 4 discussed the overall aim of this study and its limitations. Finally, in Section 5, we concluded.

## 2. Materials and Methods

### 2.1. Dataset

The database used in this study were acquired from the Imorphics dataset which is a subset of the public Osteoarthritis Initiative (OAI) database [49]. OAI that sponsored by the National Institutes of Health was initiated to maintain a natural history database for osteoarthritis that contains clinical evaluation data, radiographic (X-ray and MRI) images, and other information. It includes a multi-center that recruited 4796 men and women (ages 45–79 years) with or at risk for knee OA. The OAI’s overarching goal was to provide public resources to promote a better understanding of OA initiation and progression, which is one of the leading causes of disability in adults.

Our database contains 99 cases of 3D knee MRI dual-echo steady-state (DESS) sequences that turn up to be 15,840 total DICOM images. It covers all OA severity levels. Each case consists of 160 2D slices with the original image size of 384 × 384 pixels. Each slice with bone structures was manually segmented and served as the ground truth in this study.

### 2.2. Deep Convolutional Networks

Convolutional neural networks (CNNs) [50] are a form of artificial neural networks which are designed to identify patterns and have the ability to extract features through backpropagation from image pixels directly. A typical CNN is composed of convolutional layers, as well as other types of layers like pooling and fully connected layers. Each neuron in the convolutional layer is linked to a small local region of the input image, similar to the human visual system’s receptive field. Different neurons respond to different local regions of the input image, which overlap to provide a more accurate visual representation of the image. They have the ability to detect patterns that are undetectable by hand-crafted features. The feature extraction is done by the convolutional and pooling layers, while the fully connected layer translates the extracted features into the final output. Recent studies in pattern recognition and computer vision have revealed that CNNs are capable of solving crucial tasks such as classification, object detection, and segmentation with state-of-the-art results [51,52]. When given enough labeled data, CNNs can effectively create an exceptional hierarchical representation of the raw input images and achieve excellent results on computer vision tasks in the majority of cases. However, when CNNs are used to solve problems in the medical field, the small number of available data is a stumbling barrier to building a decent model.

### 2.3. U-Net

U-net [32] is a convolutional neural network architecture with a unique ‘U’ shape that was designed originally for the segmentation of biomedical images. As illustrated in [32], the architecture of the U-net is normally made up of an expansion path on the right side and a contraction path on the left side. The contraction path on the left side follows the structure of a common convolutional network. It comprises two convolution layers with a 3 × 3 filter size. A 2 × 2 max pooling operation with stride 2 is utilized for down-sampling of each layer. Moreover, every layer is followed by a rectified linear unit (ReLU). This path is responsible to reduce feature maps size and extract high-level features, while the expansion path on the right side comprises up-sampling layers which increase the feature maps size, feature map concatenation, and two 3 × 3 convolutional layers which refine the feature maps prepare them for the output. Finally, in order to generate the segmented output, a 1 × 1 convolution layer is utilized to map the 64D feature vectors into a determined number of classes as a final output.

**Figure 1:**
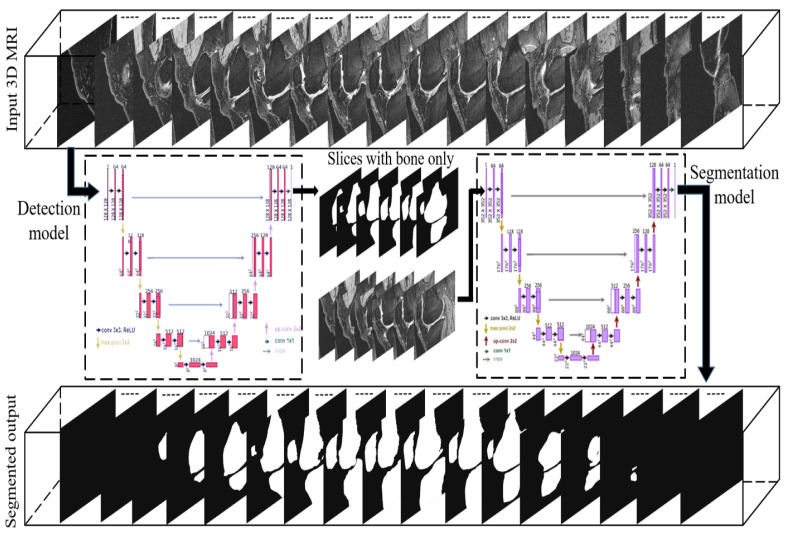
Flowchart of the proposed method.

In this study, we utilized two modified U-net models for different sub-tasks to achieve a fully automatic segmentation method for 3D knee MRI. The first model was to detect the starting and ending bone slices of the 3D MRI sequence, while the second model was to segment the bone structures from the slices between starting and ending slices. The method takes the whole MRI sequence as input, discards the slices without bone by the detection model, and then segments bone structures by the segmentation model. The flowchart of the proposed method is depicted in Figure 1, which includes the two U-net models as the core. Each knee MRI is a continuous scan sequence that consists of 160 slices. The starting and the ending of the different compartments of the bone appearance in each case occur at different slice numbers (e.g., for the same case, the femur bone starts at slice #22 and ends at slice #134, the tibia bone starts at slice #30 and ends at slice #136, and the patella bone starts at slice #50 and ends at slice #115) and those numbers very for different cases. We found that training the segmentation model with the slices that have bone appearance only can improve the segmentation results. Thus, bone detection is a necessary step to automate the whole method. In our experiments, we trained the first model (detection model) with the whole MRI sequence (slices with and without bone) to identify the slices with bone appearance and trained the second model (segmentation model) with bone slices only to obtain the accurate segmentation of bone structures. As shown in the flowchart, the output of the detection model is fed into the segmentation model.

Several improvements have been made to the original U-net in order to solve our problem. First, padding was used in our models to regulate the image size shrinkage after each convolution which keeps the output image size the same as the input image size. In the original U-net [32], no padding was used in any of the convolution layers, thus pixels near the boundaries were lost after each convolution. Second, the original study utilized the stochastic gradient descent optimizer, and here we used the Adam optimizer, a more effective optimization approach that has been used in many contemporary models [53]. Finally, we changed the activation function from softmax to sigmoid and loss function from cross-entropy to the binary cross-entropy in our detection model; and changed the loss function to soft DICE (negative DICE) in our segmentation model, which is a more effective loss measurement that improves the segmentation performance. Besides the above difference, the structures of the two models are the same; each of them requires 31,030,593 parameters to be equipped and consists of a total of 23 convolutional layers.

### 2.4. Generation of Training and Testing Sets

**Figure 2:**
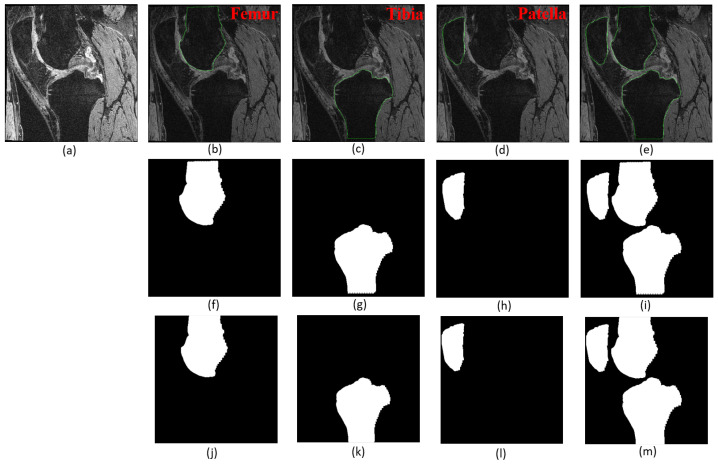
Ground truth labeling and pre-processing. (**a**) Raw image. (**b**–**e**) Manual segmentation of femur, tibia, and patella bones, respectively, and the combined. (**f**–**i**) Mask images generated from manual segmentation. (**j**–**m**) Mask images after cropping.

To prepare the data for our model, we randomly divided the 99 cases into three groups which are training, validation, and test sets. The training set includes 70% of total knee cases, i.e., 69 cases, while each of the testing and validation sets contains 15% of total knee cases, i.e., 15 cases. The testing set has not been used until the end of the study. All the slice images from the same MRI knee scan were placed in the same set since the sets were separated at the case level. A total of 11,040 slices (2D images) from 69 knees have been used as the training set for all the models while each of the testing and validation sets contains 2400 slices. Figure 2a shows an example of the raw DICOM images in our database. The femur, tibia, and patella bones were manually segmented for all slices, as shown in Figure 2b–d, respectively, while Figure 2e shows the manual segmentation of the whole knee bones together. After the manual segmentation of all DICOM slices, the binary mask images have been generated using a MATLAB script, as appeared in Figure 2f–i, using as the ground truth. All original raw DICOM images have been paired with their respective mask images. As clearly can be seen in Figure 2b–e that the manual labeling could not reach the very top and bottom of the images, thus to make sure that training data was accurate, a cropping operation has been added to the data preparation process. All images (raw and mask) were cropped 16 pixels from all sides (top, bottom, left, and right) as illustrated in Figure 2k–n to guarantee the bone begins at the image’s very top and bottom edge. After performing the cropping operation, both the original raw images and masks were adjusted from 384 × 384 pixels to 352 × 352 pixels.

### 2.5. Implementation

For implementation, Keras [54] and TensorFlow as a backend engine [55] in Python 3.7 were utilized. A computer outfitted with a NVIDIA GeForce GTX1080 Ti graphics processing unit (3584 GPU cores) has been used to carry out all experiments. All segmentation and detection models were trained using the Adam optimizer method. The dice coefficient (DICE) [56] has been used to measure the accuracy of the segmentation process, while the true positive rate of the corrected bone detection has been used to measure the accuracy of the detection process. Furthermore, the soft DICE has been used as a loss function for all segmentation models while the binary cross-entropy has been used as a loss function for all detection models in order to backpropagate through the CNN. The batch size has been set to be 16 and the learning rate was set to be 10^−5^ for all models. Keras’s callback function called EarlyStopping has been used to save the training time and to avoid model overfitting. It was responsible to stop the training process if there is no improvement of the accuracy function that used after a specified number of epochs, i.e., 30∼40. All of the experiment models were initially programmed to run for 300 epochs. For efficiency of network training time, all images and their corresponding masks were resized to 128 × 128 pixels for all the detection models but kept the original resolution 352 × 352 for segmentation models for accuracy.

## 3. Results

### 3.1. Evaluation Metrics

Different metrics were employed for each task. In terms of the detection task, we used precision, recall (also known as sensitivity), and the overall accuracy which can be calculated as follows:

(1) $$\mathrm{Recall}=\frac{\mathrm{TP}}{\mathrm{TP}+\mathrm{FN}}$$

(2) $$\mathrm{Precision}=\frac{\mathrm{TP}}{\mathrm{TP}+\mathrm{FP}}$$

(3) $$\mathrm{Accuracy}\;(\%)=\frac{\mathrm{TP}+\mathrm{TN}}{\mathrm{TP}+\mathrm{TN}+\mathrm{FP}+\mathrm{FN}}$$

where TP (true positive) is defined as bone exists in the ground truth and is detected by the model, TN (true negative) means that there is no bone in the ground truth and no bone is detected by the model as well—both ground truth mask and model’s output are pure black images. On the other hand, FP (false positive) is defined as bone is detected by the model but there is no bone appearance in the ground truth while FN (false negative) means there is bone in the ground truth, but the model does not detect that.

In the medical image segmentation tasks, a metric known as the overlap index, also known as dice coefficient (DICE) [56] is the most common metric used. DICE is computed by directly comparing the binary mask of the ground truth with that of the automated segmentation. The DICE is also used as a validation measurement of repeatability for manual segmentation in MRI, i.e., when the exact MRI image is segmented several times by the same person or different persons, the pair-wise overlap of segmentations is computed to validate the repeatability. As shown in Equation (4), the value of DICE is between 0 and 1; a perfect match is represented by 1, whereas no overlap is represented by 0. For each MRI case, we calculated the DICE score at the case level, not slice level, considering each bone compartment as a 3D object represented by the whole MRI sequence.

Several area error metrics have been calculated in addition to DICE in order to comprehensively assess the proposed segmentation approach. The similarity (SI) is determined in Equation (5) which is a general measure of how similar the automated segmentation and ground truth are. True positive ratio (TPR), false positive ratio (FPR), and false negative ratio (FNR) are described in Equations (6)–(8), respectively.

(4) $$\mathrm{Dice}=\frac{2\times \left|S_{g}\cap S_{m}\right|}{\left|S_{g}\right|+\left|S_{m}\right|}$$

(5) $$\mathrm{SI}=\frac{\left|S_{g}\cap S_{m}\right|}{\left|S_{g}\cup S_{m}\right|}$$

(6) $$\mathrm{TPR}=\frac{\left|S_{g}\cap S_{m}\right|}{\left|S_{g}\right|}$$

(7) $$\mathrm{FPR}=\frac{\left|S_{g}\cup S_{m}-S_{g}\right|}{\left|S_{g}\right|}$$

(8) FNR=1−TPR

**Figure 3:**
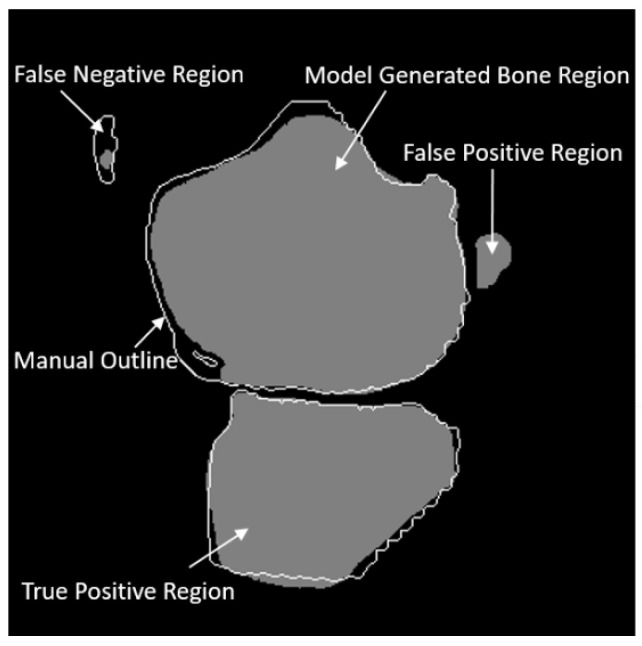
Illustration of true positive, false positive, and false negative regions.

In the above formulas, the set of bone pixels from the ground truth is denoted by $S_{g}$ whereas the set of bone pixels from the automated segmentation is denoted by $S_{m}$. Both $S_{g}$ and $S_{m}$ are the pixel sets for the whole sequence. Operator |A| means the size of set A. The TP, FN, and FP regions have been illustrated in Figure 3.

### 3.2. Experiments

In this section, we first evaluated the accuracy of the bone slice detection models, and then used the detection output to evaluate the segmentation models. Individual models were trained for each bone compartment, i.e., tibia model, femur model, patella model, plus the model for all three bones. Four detection models and four segmentation models were trained in total. The performance of these models on the testing set is reported in this section.

#### 3.2.1. Bone Slice Detection Performance

Table 2 summarized the performance of our detection models on the testing set. The testing set contains 15 cases with 2400 slices in total. The definitions of FP, FN, TP, and TN can be found in the previous section. As Table 1 shows, the overall detection accuracies of all four models are above 98%. In terms of tibia bone, our model correctly detected 1679 out of 1687 slices that contain tibia bone and missed only 9 slices as false negatives. 692 out of 712 slices that do not contain tibia bone appearance were detected while the model mis-detected bone in 20 slices that actually do not contain tibia bone.

Similar performance was obtained from the femur model while the patella model has less FP but more FN than the other two models because of the distribution of training samples. For tibia model and femur model, the majority of slices contain bone and are positive samples, therefore, the models are good at recognizing positive samples; for patella model, because the patella bone is smaller and there are fewer positive slices, the model is prone to make a negative prediction. However, the accuracies of all three models are consistent and the highest is from the model for femur bone.

The whole knee model, which is trained to detect all three knee bone compartments, achieved an overall accuracy of 98.79% and had similar FP and FN numbers as the tibia and femur models. This shows we can achieve comparable detection performance by training a single model other than three separate models for knee bone detection task.

#### 3.2.2. Segmentation Performance

**Figure 4:**
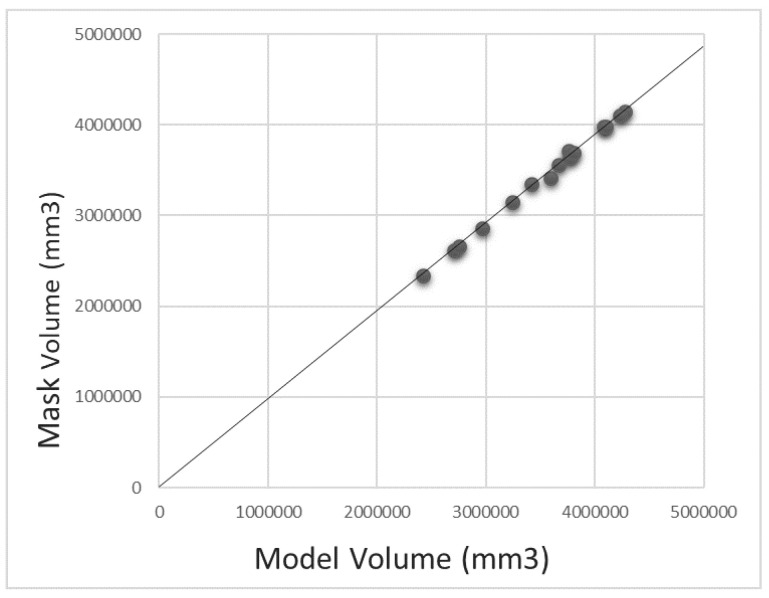
Plot comparing the manual segmentation and the proposed model’s segmentation.

**Figure 5:**
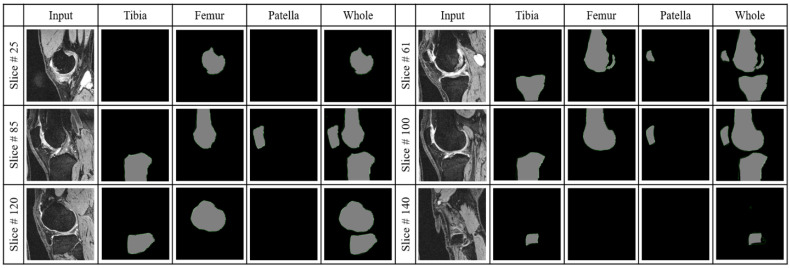
The output of the four segmentation models at different positions form an example case.

Table 3 summarized the performance of the four segmentation models on the testing set using the results from the detection models. The output of each of the detection models was fed into the corresponding segmentation model. The output of the segmentation models was evaluated using the manually labeled ground truth of bone regions. Here we calculated DICE as well as several other metrics for a comprehensive evaluation. As Table 3 shows, the average DICE reached 96.83% for tibia segmentation and 97.92% for femur segmentation. The patella model showed a lower performance with DICE 92.83%. The reason is the patella bone has a smaller size than the tibia and femur bones; therefore, the model is more sensitive to mistakes. Finally, the whole knee model that segmented the three bones at the same time reached DICE 96.94%. Figure 4 plots the bone volumes (obtained by adding up all the bone pixels from all slices in a case multiply the voxel size) of the manual segmentation versus that of our fully automatic method for the 15 testing cases. The correlation between the volume measured from manual segmentation and the proposed automatic method using Pearson’s R2 is 0.998 and the slope is 0.98 which indicates that the proposed segmentation method can systematically estimate the volume. Figure 5 depicts the models’ output for one example case at different positions. The output of the tibia model, femur model, patella model, and the whole knee model are listed in different columns.

#### 3.2.3. Ablation Study

Since the proposed method is composed of two steps in sequence, bone slice detection and segmentation, we want to study how the detection step affects the segmentation result. In this experiment, we replaced the automatic bone detection model with manual detection, i.e., we fed the segmentation models with the manually selected slices with bone. Table 4 elaborates the performance of the segmentation models on the testing set using the manually selected slices with bones only. Comparing Table 3 and Table 4, we can see that the segmentation results are slightly higher when using the manual detection as input than using the automatic bone detection results as input, however, there is no statistically significant difference between the two groups of results (see Table 5).

**Figure 6:**
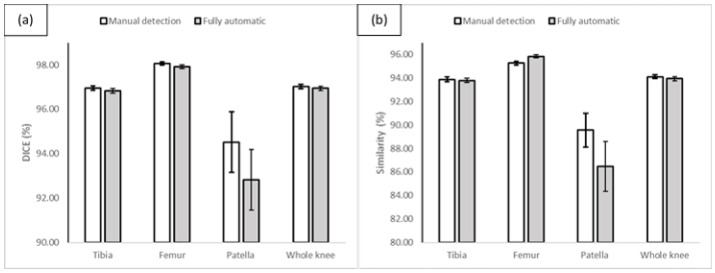
The comparison of the performance of the fully automatic models and the segmentation models using manually selected bone slices in terms of DICE (**a**) and similarity (**b**) scores.

To determine if there is a significant difference between the results, the student’s *t*-test has been carried out in terms of DICE and similarity. Table 5 illustrated the *p*-value for all the experiments. The *t*-test results indicate that there is no significant difference at the *p* = 0.05 significance level for all models in terms of DICE and similarity which proves the effectiveness of the automatic detection models. Figure 6 demonstrates a visual comparison between the two groups of segmentation models in terms of DICE and similarity scores.

#### 3.2.4. Whole Knee Model versus Individual Models

In the previous experiments, we trained separate models for each bone compartment as well as the whole knee model that can segment all three bone compartments at the same time. We want to study the difference between the result from the whole knee model and that from the combination of three individual models through post-processing. Table 5 shows the comparison between the two methods. As Table 6 shows, there is a slight improvement by training three individual models than training a single model, which reached 97.20% of average DICE and 94.55% of average similarity. The result corresponds to common sense that training a tailored model for each sub-task separately can achieve better overall accuracy, while the cost is the hassle of training multiple models and putting the results together through post-processing. Considering the slight improvement, the single whole knee model struck a good balance between efficiency and accuracy.

#### 3.2.5. The Proposed Model versus Other State-of-the-Art Models

To further validate the performance of the proposed model, a comparison has been done with other existing state-of-the-art deep learning methods including U-net [32], SegNet [57], and FCN-8 [58]. Table 7 summarized the performance of all models on the same testing set in terms of whole knee bone segmentation. As the results showed, the proposed model outperformed the other three models. All models were trained using the original image size and without any pre-processing or post-processing involved. The FCN-8 model achieved a better result than the original U-net and SegNet models in terms of the average DICE = 94.60% and an average similarity = 89.77%. On the other hand, the original U-net had a good performance on detecting the true positive regions with TPR = 99.67%. However, the false positive rate is high at the same time (FPR = 14.08%) which indicates the model included many non-bone regions as bone. The lowest performance was achieved by the SegNet model, which reached 82.49% of average DICE and 70.96% of average similarity. Comprehensively, the proposed method achieved the best segmentation accuracy in both metrics SI and DICE.

**Figure 7:**
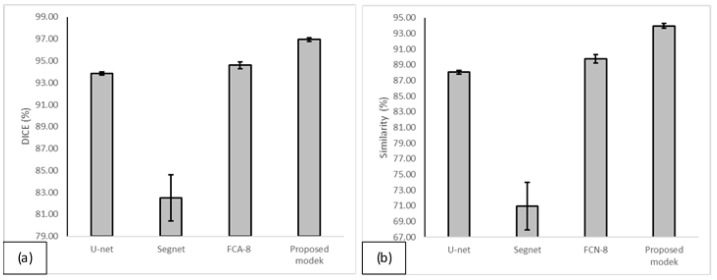
The comparison of the performance of the proposed models with the other state-of-the-art models in terms of DICE (**a**) and similarity (**b**) scores.

Moreover, in order to determine if there is a significant difference between the results of the proposed model and the other models, the Student’s *t*-test has been conducted in terms of DICE and similarity. Among the three referenced methods, FCN-8 has better performance than U-net and SegNet, so here we conducted Student’s *t*-test for FCN-8 and the proposed method. The *t*-test results indicate that there is a significant difference at the *p* = 0.05 significance level. In other words, the proposed method significantly outperformed the FCN-8 method. Table 8 illustrated the *p*-values for the Student’s *t*-tests. In addition, Figure 7a provides a visual comparison between the proposed model and the other models in terms of DICE score while Figure 7b provides the same visual comparison in terms of similarity score.

## 4. Discussion

The overall aim of this study is to develop a fully automatic method that can take the whole MRI sequence (160 slices) as input and output all the segmented bone structures. The bone segmentation can serve as the critical step for segmenting other knee structures such as cartilage which may need the extraction of the bone boundary from MR imaging sequences to facilitate the detection and segmentation of cartilage.

This study has several limitations. First is the small data set. Data labeling is time-consuming for segmentation tasks, especially the manual delineation of different bone compartments for 3D MRI image sequences. This prevented us from including more data in this study. In the future, we plan to utilize unsupervised learning or semi-supervised learning to facilitate the handling of large datasets. Second, the proposed method was evaluated using the MRI DESS sequences and has not been evaluated using other MRI sequences, such as the IWFS sequence. The generalizability of the proposed method needs further validation. Third, we have a failure case. We noticed that the testing set included one case that had many false positive regions in multiple slices of the sequence. This case was included in the evaluation and dragged down the overall performance. We need to examine and study this failure case in detail to improve the design of the proposed method and its performance.

## 5. Conclusions

This study proposed a fully automatic method to detect and segment the bones in the sequence of 3D knee MRI using modified U-net models. All bones in a knee joint including tibia, femur, and patella, are segmented. From the public OAI database, 99 cases (15,840 total DICOM images) of 3D knee MRI sequences have been used in this study. Without any human intervention, the trained system takes the whole 3D MRI sequence with 160 slices as input, detects the slices with bone, and outputs the segmentation results for these slices. The bone slice detection model accomplished an accuracy of 98.79% on the testing set which prepared the segmentation model well to delineate the whole knee bones. The segmentation model achieved DICE 96.94% and similarity 93.98% on the testing dataset for whole knee segmentation. We further conducted an ablation study which proved the effectiveness of the detection model, and a comparison study which showed that the single whole knee segmentation model struck a good balance between efficiency and accuracy. In addition, we compared the proposed method with several other state-of-the-art segmentation methods including U-net, SegNet, and FCN-8. The proposed model outperforms the other three methods in both DICE and similarity score.

One of future work is to improve the segmentation accuracy of patella bone which had lower accuracy than other bones. Besides, the bone segmentation result can be used as an initial step to detect and segment other knee structures and biomarkers, including cartilage, effusion, bone marrow lesion, and meniscus. Direct segmentation of these structures without bone identification is a more challenging task due to the small and complex structures.

## Figures and Tables

**Table 1:** Summary of knee image segmentation methods.

Paper	Year	Approach	Dataset	Region of Interest	Performance	Advantages	Drawbacks
Wu, et al. [47]	2014	MSL, SSM, Graph cut	465 CT scans	FB, TB, PB, FiB	AvgD: FB-0.82 mm, TB-0.96 mm, PB-0.68 mm, FiB-0.96 mm	High accuracy of overlap removal for bones	Boundary leakage
Fabian et al. [48]	2015	Random forest classifier	20 MRI	FB	DICE: 92.37% Sens: 91.75% Spec: 99.29%	Short training time	Smaller dataset used, classification accuracy relied heavily on the quality of labeled data
Liu et al. [45]	2018	SegNet, 3D deformable model	100 MRI (SKI10)	FB, FC, TB, TC	AvgD: FB-0.56 mm, TB-0.50 mm, VOE: FC-28.4%, TC-33.1%	Low computation cost, short training time	Compared SegNet with only U-Net
Liu [46]	2018	R-Net	60 MRI (SKI10), 2 clinical MRI datasets	FB, FC, TB, TC	DICE: FB-97.0%, TB-95.0%, FC-81.0%, TC-75.0%	The first study to translate one MRI sequence to another	No comparison with other techniques
Ambellan et al. [44]	2019	U-net, SSM	100 MRI (SKI10), 88 (OAI Imorphics), 507 (OAI-ZIB)	FB, FC, TB, TC	DICE: FB-98.6%, TB-98.5%, FC-89.9%, TC-85.6%	Achieved good segmentation accuracy, time-efficient	Compromise between memory and size for choosing subvolume to train 3D CNN

**Table 2:** The performance of the detection models on the testing set.

	FP	FN	TP	TN	Recall	Precision	Accuracy (%)
Tibia	20	9	1679	692	0.995	0.988	98.79
Femur	20	8	1786	586	0.996	0.988	98.83
Patella	9	28	950	1413	0.971	0.992	98.46
Whole Knee	20	9	1831	540	0.995	0.989	98.79

**Table 3:** The performance of the segmentation models based on the detection results on testing set.

	TPR (%)	FPR (%)	FNR (%)	SI (%)	DICE (%)
Tibia	96.93	3.27	3.07	93.87	96.83
Femur	98.26	2.46	1.74	95.91	97.92
Patella	96.45	11.50	3.55	86.61	92.83
Whole Knee	98.51	4.83	1.49	93.98	96.94

**Table 4:** The performance of the segmentation models using the manually selected bone slices.

	TPR (%)	FPR (%)	FNR (%)	SI (%)	DICE (%)
Tibia	97.78	3.99	2.23	94.03	96.96
Femur	97.69	2.09	2.31	95.25	98.06
Patella	95.37	6.36	4.63	89.71	94.52
Whole Knee	98.60	4.74	1.40	94.14	97.02

**Table 5:** The comparison of the fully automatic segmentation results and the segmentation results using manually selected bone slices.

	With Manual Detection		With Automatic Detection		*p*-Value	*p*-Value
	SI (%)	DICE (%)	SI (%)	DICE (%)	(DICE)	(SI)
Tibia	94.03	96.96	93.87	96.83	0.400	0.729
Femur	95.25	98.06	95.91	97.92	0.399	0.330
Patella	89.71	94.52	86.61	92.83	0.304	0.239
Whole Knee	94.14	97.02	93.98	96.94	0.489	0.499

**Table 6:** The comparison of one vs. three segmentation models for whole knee segmentation.

	TPR (%)	FPR (%)	FNR (%)	SI (%)	DICE (%)
Whole knee model	98.60	4.74	1.40	94.14	97.02
Combination of three models	97.66	03.29	02.34	94.55	97.20

**Table 7:** The performance of testing set of the proposed model and other state-of-the-art models for whole knee segmentation.

	TPR (%)	FPR (%)	FNR (%)	SI (%)	DICE (%)
U-net	99.67	14.08	0.33	87.38	93.26
SegNet	83.17	20.16	16.83	70.96	82.49
FCN-8	92.66	3.20	7.34	89.77	94.60
Proposed Method	98.51	4.83	1.49	93.98	96.94

**Table 8:** The comparison of the proposed model vs. FCN-8 models for whole knee segmentation.

Proposed Method		FCN-8		*p*-Value	*p*-Value
SI (%)	DICE (%)	SI (%)	DICE (%)	(DICE)	(SI)
93.98	96.94	89.77	94.60	0.0000077	0.0000069

## Data Availability

Not applicable.
